# An update on inborn errors of V(D)J recombination

**DOI:** 10.1152/physiol.00004.2026

**Published:** 2026-03-28

**Authors:** Ina Schim van der Loeff, Manisha Ahuja, Rui Chen, Eleonora Patane, Sophie Hambleton

**Affiliations:** 1 Newcastle University Translational and Clinical Research Institute, Newcastle upon Tyne, UK; 2 Paediatric Immunology and Infectious Diseases, Great North Children’s Hospital, Newcastle upon Tyne Hospitals NHS Foundation Trust, Newcastle upon Tyne, UK; 3 Department of Immunology, Newcastle Hospitals NHS Foundation Trust, Newcastle upon Tyne, UK; 4 Messina University, Department of Pediatrics, Messina, Italy

**Keywords:** RAG, NUDCD3, Omenn syndrome, V(D)J recombination, inborn errors of the immune system

## Abstract

V(D)J recombination is the fundamental process by which developing T and B lymphocytes generate diverse antigen receptors, enabling adaptive immunity. This tightly regulated program operates exclusively in lymphoid precursors during G1 phase and depends on the lymphocyte specific RAG1–RAG2 recombinase to introduce programmed DNA double-strand breaks at recombination signal sequences, followed by repair through the classical non-homologous end-joining (c-NHEJ) pathway. Disruption of any step in this molecular choreography compromises antigen receptor diversity and underlies a spectrum of inborn errors of immunity (IEI), ranging from severe combined immunodeficiency (SCID) to immune dysregulation with autoimmunity and granulomatous disease.

In this review, we place disorders of V(D)J recombination within the broader framework of T-cell development, detailing the temporal waves of recombinase activity, chromatin accessibility, and DNA damage responses that guide thymocyte differentiation. We discuss pathogenic variants affecting the cleavage phase (RAG1, RAG2, and the recently identified RAG co-chaperone NudC domain-containing 3, NUDCD3), end processing (ARTEMIS), ligation and repair (LIG4, XLF, XRCC4, PRKDC), and genome surveillance pathways (ATM, MRN complex, RNF168), highlighting genotype–phenotype correlations and mechanisms driving immune deficiency and dysregulation.

We briefly review recent diagnostic advances, including newborn screening using T-cell receptor excision circles, repertoire sequencing, and functional assays, alongside current therapeutic strategies. Finally, we outline key unanswered questions and argue that continued integration of clinical observation with molecular discovery is essential to improve outcomes and deepen understanding of adaptive immune development.

## Introduction

V(D)J recombination is a carefully orchestrated molecular process through which T and B lymphocytes (“cells”) generate unique and diverse antigen receptors, a requisite step in their development as well as their ultimate function. Both T and B cells express surface-bound antigen receptors but B cells are uniquely able to secrete their BCR as immunoglobulin when activated. Thus, V(D)J recombination occurs at seven loci: the immunoglobulin heavy chain, κ and λ light chain loci in B cells and the TCR α, β, γ and δ loci in T cells.

**Figure 1:**
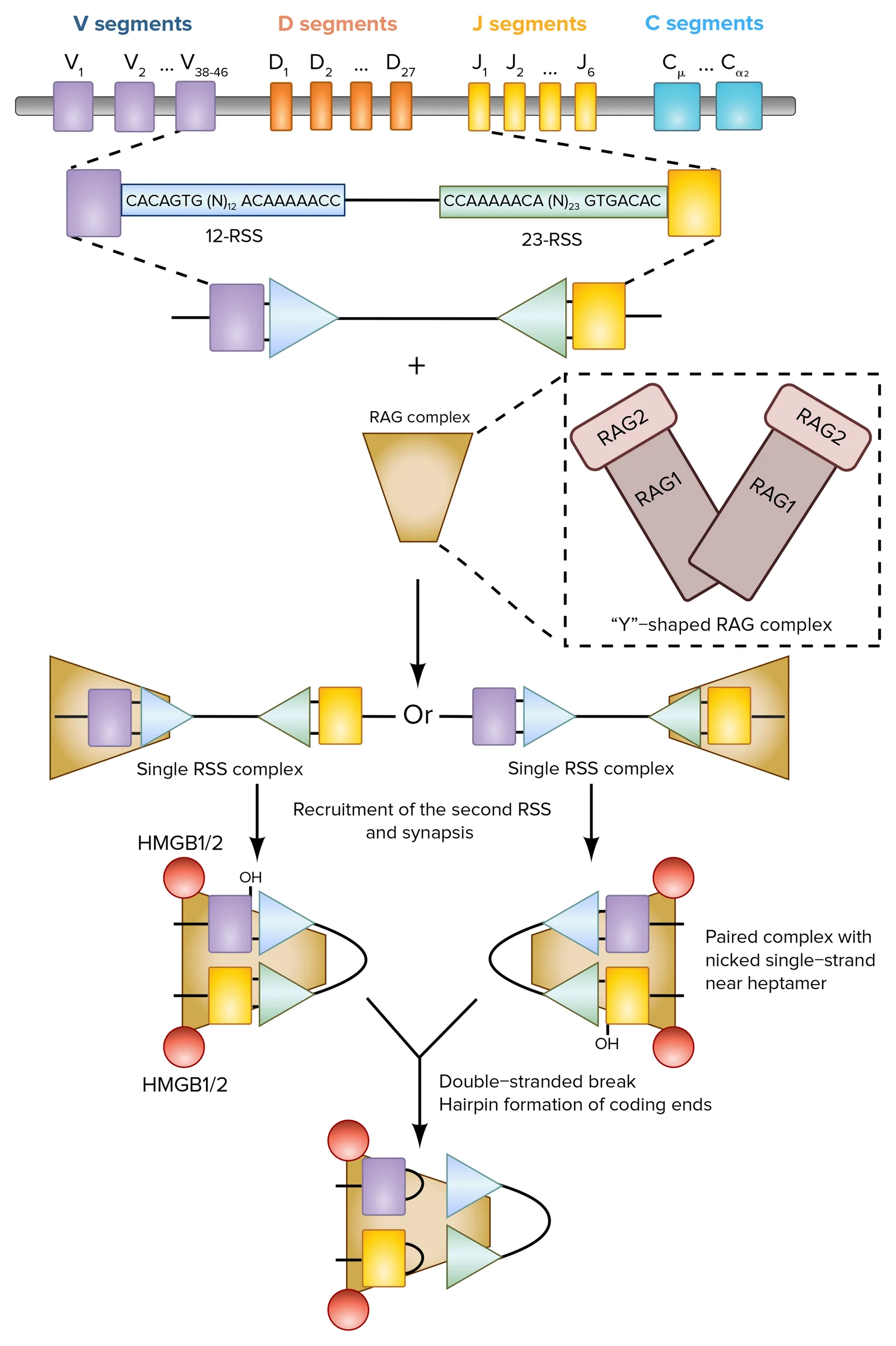
Schematic illustrating the creation of DNA double strand breaks by RAG recombinase during V(D)J recombination. Created with Biorender

**Figure 2:**
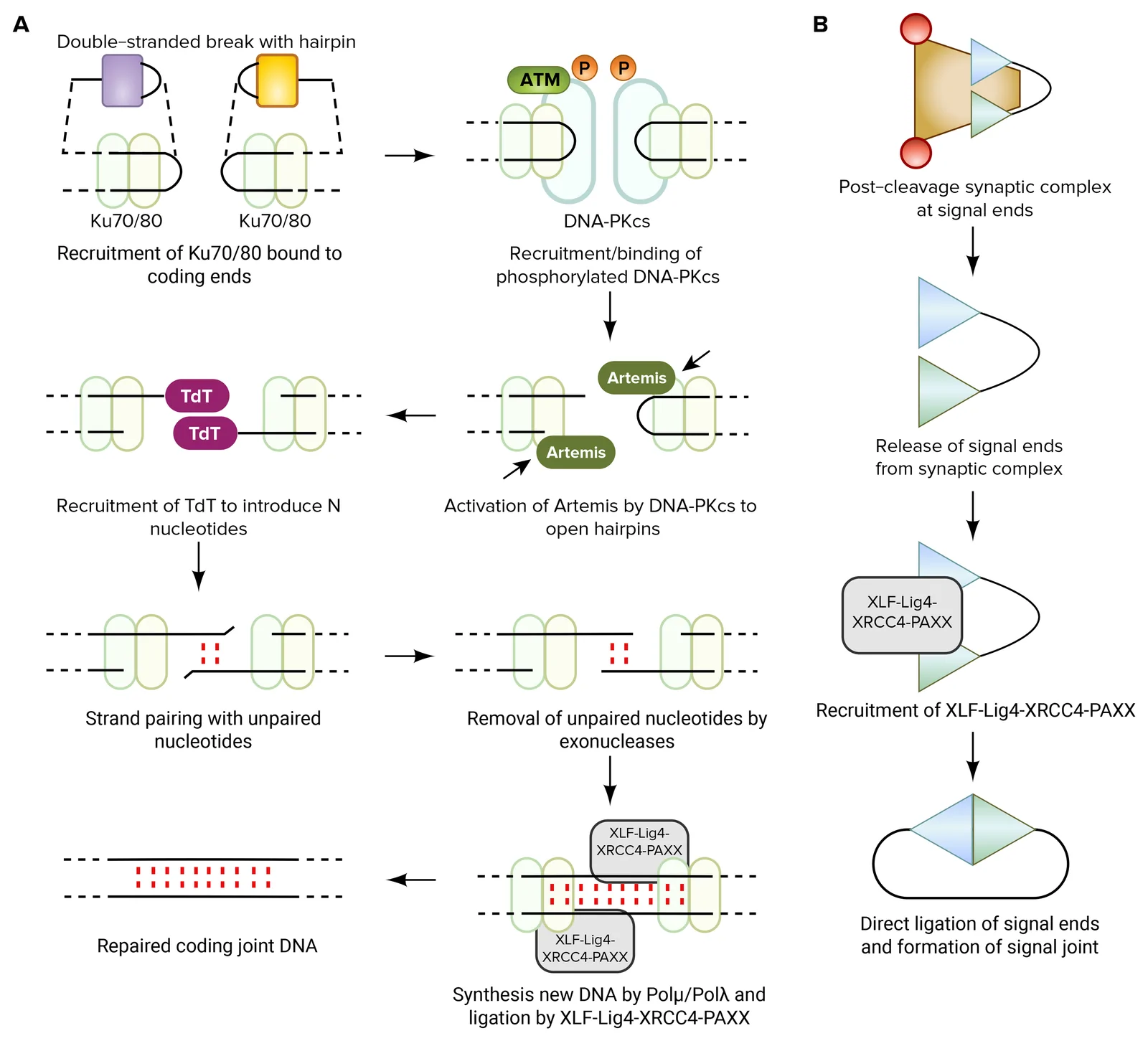
Schematic illustrating the repair of DNA double strand breaks by NHEJ machinery during V(D)J recombination. Created with Biorender

V(D)J recombination occurs only in developing lymphocytes at the G1 phase of the cell cycle and acts on antigen receptor gene loci that feature multiple variable (V) and joining (J) gene segments and, in the case of TCR δ and β genes, multiple diversity (D) segments too. Highly specialized machinery enables the rearrangement of germline DNA to bring together one each of the requisite gene segments, conferring combinatorial diversity among independent clones expressing the resulting transcript. As depicted in figure 1, the process is initiated by the RAG recombinase, which introduces double strand breaks (DSB) at specific recognition sequences flanking the V, D and J gene segments. Breaks are repaired through ubiquitous non-homologous end-joining (NHEJ) machinery, requiring the endonuclease Artemis as well as various other nucleases and DNA polymerases (including DNA ligase 4, XLF/Cernunnos and XRCC4; figure 2). Mismatched or damaged nucleotides are removed while the variable addition of random nucleotides by terminal deoxynucleotidyl transferase (TdT) generates additional diversity, particularly impacting the critical third Complementarity Determining Region (CDR3) of the resulting TCR(1). The signal ends excised from the recombining V, D or J gene segments are ligated to form a stable non-replicating DNA episome known as a receptor excision circle (TREC or KREC in T or B cells, respectively). The abundance of TRECs and or KRECs reflects thymic and bone marrow output of the relevant cell types and forms the basis of newborn screening for SCID.

Mendelian disorders of V(D)J recombination highlight the critical importance of antigen receptor diversity to human immunity and have elucidated novel genes and pathways essential to recombination, including most recently the role of NUDCD3 in facilitating RAG function. These disorders comprise a broad spectrum of disease associated with varying degrees of impairment of V(D)J recombination. In the complete absence of recombination activity, T and B cells fail to develop. This causes severe combined immunodeficiency (SCID), which is typically fatal in infancy without corrective therapy such as hematopoietic stem cell transplantation (HSCT). If recombination activity is only partially impaired, T and B cells do develop but are often ineffective in their protective functions, and might cause immune dysregulatory phenomena such as autoimmunity and/or granuloma formation. In the most severely hypomorphic conditions, oligoclonal T cells emerge into the periphery and can produce a severe inflammatory picture known as Omenn syndrome, characterized by faltering growth, diarrhea, eosinophilia and erythroderma as well as profound combined immunodeficiency (2).

Early diagnosis and curative therapy are key and lead to improved clinical outcomes. In fact, universal newborn screening for SCID has been introduced in many countries and results in improved survival following stem cell therapy as a result of freedom from infection and immunopathology (3). Treatment outcomes in SCID and other IEI are better if the molecular diagnosis is known(4), which enables genetic counselling in affected families and can facilitate therapeutic decision-making, particularly in isolated T cell immunodeficiency with a differential diagnosis of thymic dysfunction. Meanwhile, identifying radiosensitivity as in disorders of NHEJ is essential to titrate pre-transplant chemotherapy.

In this review, we consider inborn errors of V(D)J recombination and their associated clinical phenotypes within the broader context of T cell development.

### Coordination of V(D)J recombination within T cell development

**Figure 3:**
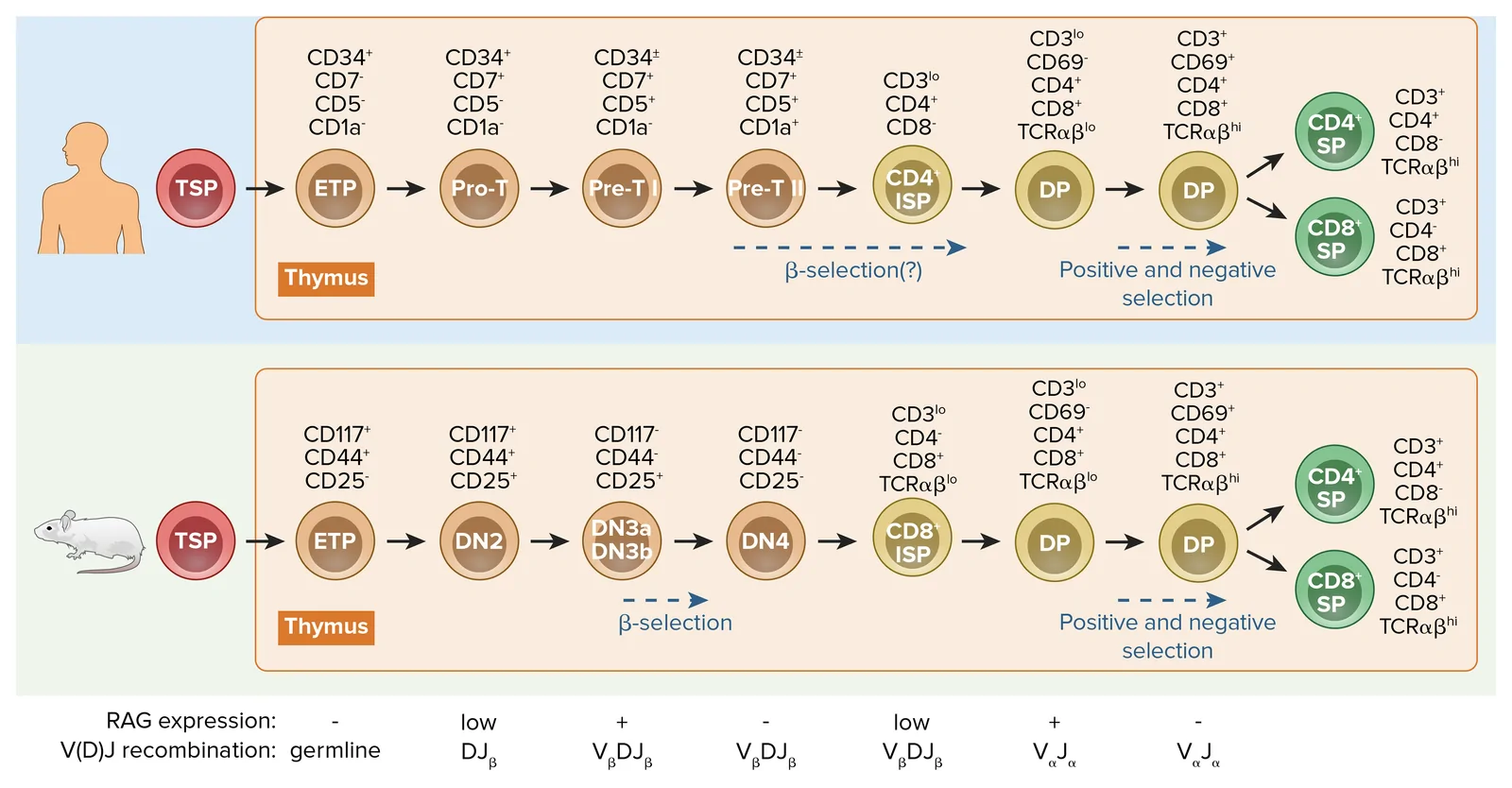
Schema comparing T cell development within the thymus of mouse and human. Early human thymocyte stages are subdivided based on (12, 167–170). Created with Biorender

T cells arise from bone marrow-derived CD34^+^ thymic seeding progenitors (TSP) which enter the thymus lacking CD4 and CD8 expression (double negative, DN) but expressing high levels of CD44 in mice, corresponding to CD34^+^CD7^-^CD1a^-^ early thymic progenitors in human (figure 3). There is evidence of transient recombinase gene expression before arrival in the thymus (5) but no full V(D)J recombination happens until later. Responding to thymic microenvironmental cues including strong Notch signaling(6), DN thymocytes traverse sequential developmental stages (7) and migrate from the cortex to the subcapsular zone. Subsequent development depends on intact signalling via both TCR and interleukin-7 receptor (IL-7R) such that T cells are generated that can survive and proliferate once they leave the thymus and are “fit” to recognize antigen without being reactive to self (8, 9). Importantly, early DN thymocytes are a heterogeneous population, with commitment to the T cell lineage only truly occurring at the CD44^-^CD25^+^ DN3 stage in mice and the CD34^+^CD1a^+^ Pre-T II stage in humans (6)(10–12).

*RAG1* and *RAG2* transcription is tightly controlled and occurs in waves, concurrent with the requirement for V(D)J recombination (13). In all organisms studied, *RAG1* and *RAG2* are located physically close to one another and transcribed convergently suggesting they might have arisen evolutionarily as a single transposable element. Distinct lineage-specific *cis*-regulatory elements direct these waves of *RAG* expression (14, 15). For example, the enhancer *Erag* (enhancer of *Rag*), upstream of *Rag2*, regulates *Rag* expression in B cells (16). An anti-silencer element (*ASE*), also upstream of the *Rag2* promoter, regulates T cell *Rag* expression (17). The transcription factor E2A is indispensable for both T and B cell development as well as *Rag1* and *Rag2* expression and a recent study mapped its binding to a T cell specific enhancer, Rag T-cell enhancer (*R*-*Ten*) which overlaps with ASE and two B cell specific (*R1B* and *R2B*), one of which overlaps with the previously identified *Erag* (18). In both cell types, *Rag* expression is repressed by tonic signalling downstream of non-autoreactive, successfully rearranged antigen receptors.

In T cells, the first wave of RAG expression coincides with the DN2-3 stage in mice, CD34^+^ CD7^+^ cells (Pro-T to Pre-T) transition in humans, enabling rearrangement of the *TRG, TRD* and *TRB* loci (19). Should surface expression of TCRγ and δ chains occur, these deliver a strong TCR signal that diverts cells to the γδ fate and prevents ongoing expression of RAGs, bringing TCR recombination to an end (20–22). More frequently, however, these genes are non-productively rearranged, and recombination of the *TRB* locus proceeds. Productive rearrangement and ensuing TCRβ expression alongside an invariant pre-TCRα (pre-TCR) allows T cells to surmount the β-selection checkpoint which critically depends on both Notch and pre-TCR signalling in the mouse (23). T cells that fail to receive a suitable positive selection signal via the pre-TCR are lost by programmed cell death. Pre-TCR signalling inhibits further rearrangement of the *TRB* locus, known as allelic exclusion (24), and enables upregulation of CD4 and CD8 to produce DP thymocytes.

The second wave of *RAG* expression occurs during this DP stage and enables the *TRA* locus to rearrange, in the process losing the embedded *TRD* (23). Unlike recombination at the *TRB* locus, TCRα rearrangement occurs in an iterative and sequential fashion until a functional TCR is generated (25, 26), capable of recognising peptide in the context of individual and highly polymorphic MHC (8). The nature of this ongoing gene revision results in a lack of allelic exclusion for TCRα, reflected by mature T cells expressing dual TCRs (two TCRα chains paired with the same TCRβ) (27). Furthermore, its temporal and spatial pattern mean that inborn errors of V(D)J recombination tend to produce systematic skewing of the TCRα repertoire in a manner that can be diagnostically helpful(28). Positive selection signals through the TCRαβ ultimately downregulate RAG expression permanently (29–31) and initiate directed migration towards the medulla (32). Here, developing T cells commit to becoming either CD4 or CD8 T cells (8) and undergo negative selection to prevent self-reactive immune responses: those which recognise self antigens with high affinity are either clonally deleted by programmed cell death or diverted to the regulatory T cell lineage (33).

### Molecular players in V(D)J recombination

#### Assembly and regulation of the RAG1–RAG2 recombinase complex in T cells

**Figure 4:**
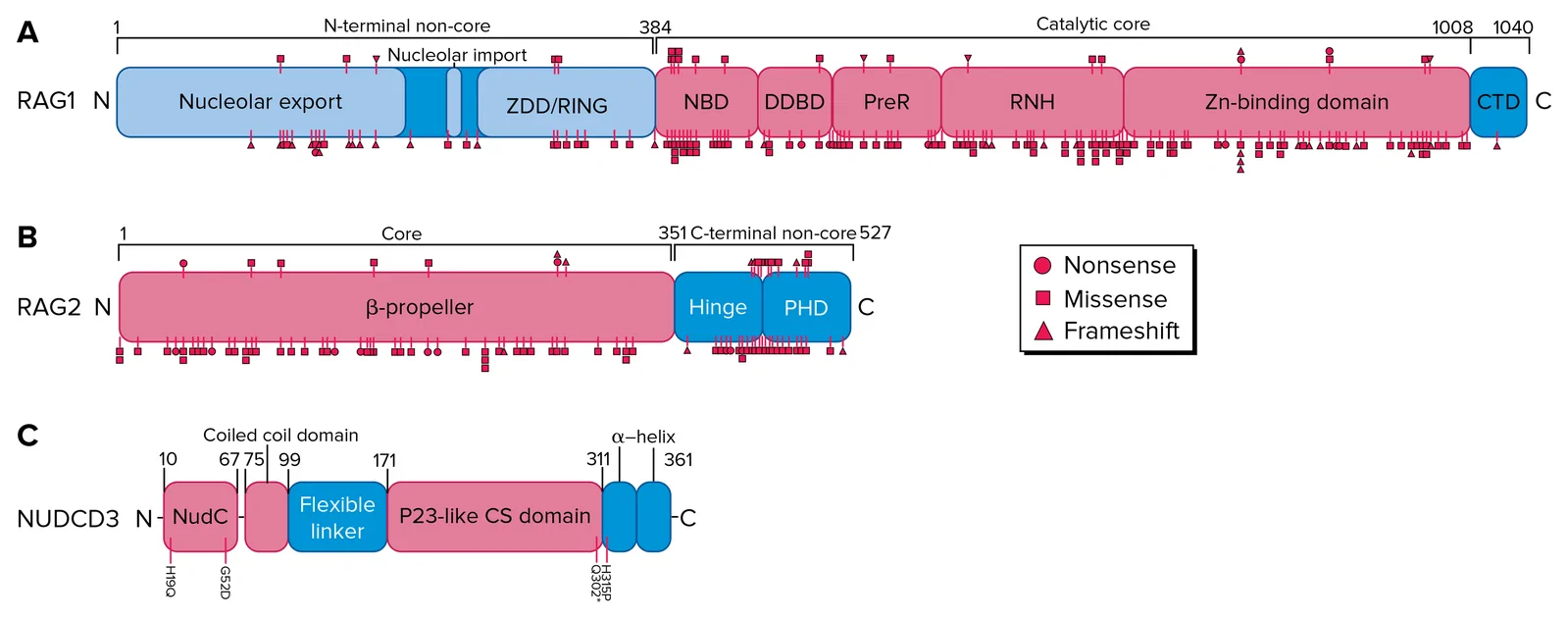
Domain structure and distribution of pathogenic variants in proteins involved in DNA cleavage during V(D)J recombination (RAG1, RAG2 and NUDCD3). ZDD/RING – zinc-binding dimerisation/RING finger domain; DDBD, dimerization and DNA-binding domain; NBD, nonamer-binding domain; PHD, plant homeodomain; CTD, carboxy-terminal domain; preR, pre-RNase H; RNH, catalytic RNase H; CS, CHORD SGT1 domain. Created with Biorender

The human *RAG1* gene is located at chromosome 11p1, encoding a single protein product that is 1043 amino acids long and contains multiple structured domains. RAG1 can be divided into three main regions, the N-terminal non-core region (aa 1-384), the core region (aa 384-1008), and the C-terminal non-core region (aa 1008-1040) (Figure 4)(34, 35). The N-terminal non-core region includes nucleolar import and export domains, which regulate RAG1 protein abundance and localization by controlling its trafficking into and out of the nucleus and nucleolus (36). Adjacent to this is the RING finger and zinc finger motif that provides histone H3 ubiquitin ligase activity and allows for homodimer formation (37).

Although regarded as non-core, the N-terminal region is required to achieve full RAG1 protein function for a normal level of V(D)J recombination activity, as well as to interact with several proteins such as those involved in NHEJ pathway (38, 39). The core region with catalytic recombinase activity consists of a nonamer-binding domain (NBD), dimerization and DNA-binding domain (DDBD), pre-RNase H (preR), catalytic RNase H (RNH), zinc-binding domain including two distinct regions with canonical cysteine and histidine zinc-binding residues (ZnC2 and ZnH2) and the carboxy-terminal domain (CTD) (34, 40). The NBD specifically recognises and binds the nonamer component of the recombination signal sequence (RSS), contributing to the sequence specificity of RAG1 activity (41). The DDBD supports DNA binding and provides an additional homodimerization interface (42). The CTD interacts with double-stranded DNA in a largely sequence-independent manner, relying on surrounding domains and motifs to confer specificity (43, 44). The ZBD, positioned across the central domain and CTD, contains two zinc-binding modules that mediate interaction with RAG2 (45, 46). Although the C-terminal non-core tail is short, it has been implicated in inhibiting hairpin formation and fine-tuning DNA binding and cleavage together with the C terminus of RAG2 (47).

The human *RAG2* gene is also located at chromosome 11p1, encoding a 527-amino acid RAG2 protein. RAG2 can be separated into two regions, a core region (aa 1-351) and a C-terminal non-core region (aa 352-527) (Figure 4) (34, 35). The core region contains six Kelch-like repeats that fold into a six bladed β-propeller, a structure that is essential for efficient DNA cleavage and for providing a stable interaction interface for RAG1 (34, 48). The C-terminal non-core region consists of an acidic hinge domain and a plant homeodomain (PHD). The core region and the PHD domain are linked by a hinge region with a high concentration of acidic residues, providing the flexibility required for recombination activity yet steering the post-cleavage complex towards DSB repair by NHEJ, thus contributing to genome stability (49). The autoinhibitory motif within the hinge restrains RAG activity unless the PHD domain binds H3K4me3, a hallmark of transcriptionally active chromatin, thereby licensing DNA cleavage (50, 51). Residues within the very C-terminus of the non-core region mediate cell-cycle regulated turnover of RAG2; phosphorylation in this region promotes recognition by the Skp2 ubiquitin ligase and degradation, thereby confining RAG2 abundance to G1 phase (52, 53).

When expressed alone, RAG1 predominantly exists as a homodimer, whereas transiently expressed RAG2 is present as a mixture of monomeric, dimeric or larger forms (54). The localisation of the RAG proteins is also tightly regulated and RAG1 is normally retained within the nucleoli pending the expression of RAG2(36). For recombination to occur, RAG1 must relocate and assemble with RAG2 in the nucleoplasm to access chromatin containing RSSs (36, 55). As discussed below, we recently reported hypomorphic variants in *NUDCD3* which undermine this relocalisation step, identifying NUDCD3 as an important co-chaperone for RAG recombinase functionality. Upon assembly, RAG1 and RAG2 form a characteristic Y-shaped heterotetramer composed of two heterodimers, with RAG1 forming the base and RAG2 positioned at the distal tips (48, 54). The catalytic centre situates at the junction, where the two arms form the Y shape (48).

Recombination activity is restricted to a permissive chromatin environment, thereby restraining the RAG complex’s otherwise potent threat to genome stability (56). The recombination process begins with recognition and binding of RSSs by RAG1 (57). Flanking V, D and J gene segments, RSSs are motifs composed of a conserved heptamer (5′-CACAGTG-3′) and nonamer (5′-ACAAAAACC-3′) separated by a less conserved spacer of either 12 or 23 nucleotides. Efficient DNA cleavage requires the heterologous pairing of RAG-bound 12- and 23-RSS to assemble a 12-23 synaptic complex, which is referred to as the “12-23 rule” to ensure the ordered pairing of D to J and followed by V to DJ segment joining(57). The “choice” of V, D and J segments for recombination is influenced by the intrinsic strength of their RSSs together with the accessibility and epigenetic state of relevant loci (58, 59).

Upon formation of the paired complex, RAG and HMGB1/2 induce a conformational change that positions the heptamer–coding junction for cleavage (60–62). RAG1 first introduces a nick on the 5′ strand adjacent to the heptamer and creates a 3′ hydroxyl group that then engages the opposite strand in a transesterification reaction (63). This two-step cleavage mechanism produces covalently sealed hairpin coding ends and blunt signal ends, with the RAG complex remaining bound in a cleaved signal complex poised for downstream end-joining repair, the second phase that completes recombination (Figure 2) (64).

#### DNA end processing and classical non-homologous end joining (c-NHEJ)

During V(D)J recombination, the repair of RAG-induced double-strand breaks (DSBs) relies almost exclusively on the classical non-homologous end-joining (cNHEJ) pathway. Following paired cleavage of recombination signal sequences by the RAG1/2 complex, two covalently sealed hairpinned coding ends (CEs) and two blunt signal ends (SEs) are produced. The transition from the RAG-bound post-cleavage complex to the NHEJ machinery during G1 phase is tightly regulated to prevent aberrant joining and safeguard genome integrity. The first step is recognition of DNA ends by the Ku70/Ku80 heterodimer, which rapidly encircles both CEs and SEs and recruits the DNA-PK holoenzyme. DNA-PKcs, once binding to CEs, triggers autophosphorylation-dependent conformational changes that allow access to Artemis. Encoded by DNA cross-link repair 1C, *DCLRE1C*, Artemis has endonuclease activity and introduces asymmetric nicks at the hairpin ends, generating short single-stranded extensions. These overhangs become substrates for terminal deoxynucleotidyl transferase (TdT), which adds non-templated nucleotides (NTN) and contributes substantially to junctional diversity in coding joints in a process known as junctional editing.

Subsequently the alignment of processed ends is mediated by XRCC4 and XLF, which form extended filaments bridging DNA ends and stabilizing a synaptic complex; the latter is the result of the close apposition of DNA ends—maintained initially by RAG proteins and later reinforced by NHEJ factors such as Ku70/Ku80, XRCC4, XLF, and DNA-PKcs. This is essential to create a protected environment that prevents end dissociation or aberrant events of joining (63, 65, 66). Besides those mentioned above, redundant accessory factors are described, such as PAXX. Its action overlaps XLF in further reinforcing end-joining and stabilizing the DNA-PK complex at breaks, and its absence has not been associated to human disease (67, 68). Ultimately Ligase IV, positioned at the repair site by the scaffold XRCC4, performs the final ligation step, completing the coding and signal joins. The exquisite coordination of these factors ensures efficient and faithful repair of programmed DSBs, and defects in some components of this machinery underlie a spectrum of immunodeficiencies marked by impaired lymphocyte development and genomic instability(68).

#### DNA damage sensing, checkpoints, and fate decisions during TCR rearrangement

ATM is another key regulator of the DSB repair response. Like DNA-PKcs, ATM has a PI3K domain making it one of three members of the Phosphoinositide-3-Kinase (PI3K)-like Kinase (PIKK) protein family (DNA-PKcs, ATM and (ATR) protein) which are all essential to the DNA DSB repair response (69). ATM is a large threonine / serine kinase which has no intrinsic DNA binding ability. It is recruited to DSBs through protein scaffolding and histone modifications (70). Specifically, ATM is recruited to DSB by the MRN complex consisting of MRE11, RAD50 and NBN (70) and is at least partly activated by autophosphorylation after which it phosphorylates nearby H2AX histones creating foci of γH2AX. This recruits MDC1 which scaffolds more ATM and MRN at DSBs creating a positive feedback loop of ATM activation and H2AX phosphorylation (71). Activated ATM then phosphorylates hundreds of substrates regulating the cellular responses to DNA damage including DNA repair, cell cycle regulation, alteration in chromatin structure and apoptosis (69). Substrates include p53 leading to cell cycle arrest in G1 and CHK2 triggering apoptosis if damage is too great (69). Importantly, ATM and MRN activation also triggers a cascade of histone ubiquitination by RNF8 and RNF168 recruiting 53BP1 to the site of DNA damage where it is phosphorylated by ATM. Activated 53BP1 inhibits homologous recombination, an alternative to NHEJ DSB repair, by preventing end resection (72).

The proteins ATM and the MRN complex recruit to DSB and γH2AX foci are thought to create a protein-chromatin network resulting in local loosening of chromatin but also tethering DSB ends together (73). In developing thymocytes, γH2AX and NBN form nuclear foci which colocalise with *TRA* (74). DSBs created by RAG are tethered in the RAG post-cleavage complex and recruit ATM. One hypothesis is that ATM, MRN, γH2AX and 53BP1 stabilise RAG-induced DSBs allowing correct end processing and ligation (73). Perhaps unsurprisingly, mice and humans share significant overlapping redundancies in the regulators of DSB repair with significantly impaired V(D)J recombination only apparent if two or more repair factors are missing. For example, ATM or XLF deficiency alone results in only a mild reduction in lymphocytes while biallelic deletion of *Xlf* and *Atm* almost completely abrogates lymphocyte development (75).

ATM clearly enforces cell cycle checkpoints through p53, CHK2 and ATR activation and promotes cell death if there are persistent unrepaired RAG DSB. In developing lymphocytes in the thymus these checkpoints come at the transition of DN3 to DN4 and TCR intermediate DP stage when *TCRB* and *TCRA* recombination occur. Consistent with this, ATM deficient mice have an increased DN3/DN4 thymocyte ratio and fewer SP thymocytes, suggesting inefficient β- and α-selection (76, 77). Interestingly, there is evidence that ATM promotes the survival of developing lymphocytes with RAG DSBs undergoing V(D)J recombination in pre-B cells by inducing PIM2 expression - this prevents proliferation of pre-B cells that have initiated V(D)J recombination and have RAG DSBs that could be aberrantly repaired. By activating the kinase PIM2, ATM has also been shown to promote the survival of developing thymocytes by inhibiting the pro-apoptotic protein BAD (78), perhaps to allow more time in specific phases of the cell cycle for physiological DSB to be repaired.

The role of ATM and 53BP1 in tethering the two ends on either side of a DSB together is perhaps more important in class switch recombination (CSR) than V(D)J recombination. During CSR the *IgHM* gene constant region is replaced by an alternative constant region to generate different Ig isotypes. During CSR and somatic hypermutation, AID deaminates cytosine bases turning it into uracil which is recognized as thymine, in other words converting a C-G base pair to a T-A. CSR occurs earlier, during initial T cell contact. This process critically depends on ATM, DNA-PKcs, NBS1 and H2AX and deficiency in any of these results in failure to switch from IgM to other isotypes (79). Interestingly, although not described in humans, 53BP1 deficiency in mice results in a CSR defect.

### Pathway-based overview of genes involved in V(D)J recombination and associated clinical phenotypes

#### Cleavage: core recombinase defects

A large number of pathogenic variants in *RAG1* or *RAG2* have been described in association with primary immunodeficiency, many of which have been tested experimentally (Figure 4; Supp Tables S1 & S2). Systematic analysis of human *RAG1* variants has established a clear relationship between biochemical recombination activity and clinical severity (80). Variants with little to no measurable activity present as classical T-B- SCID or as Omenn syndrome (OS), in which the emergence of oligoclonal, frequently autoreactive T cells drives eosinophilia, elevated IgE and pathological infiltration of tissues including the skin and viscera (2, 34, 81).

Further residual recombination activity broadens the phenotype to leaky SCID or combined immunodeficiency with granulomas and/or autoimmunity (CID-G/AI), as documented in several large international cohorts (80–83). Multi-omic profiling of patients across this spectrum confirms that hypomorphic RAG activity not only restricts repertoire formation but also promotes immune dysregulation through aberrant T-cell activation, TH2-skewing in OS, and a shared inflammatory signature across phenotypes (81). These studies emphasise that RAG deficiency is not simply a failure of lymphocyte development, but a disorder of aberrant repertoire-driven immunopathology.

*NUDCD3*, situated on human chromosome 7p13, has recently been identified as a SCID/OS-associated gene and represents a completely novel mechanism of impaired V(D)J recombination (84). NUDCD3 is a member of the NudC family of p23 domain-containing proteins, and is ubiquitously expressed in vertebrates (85). Comprising 361 amino acids, it contains a conserved N-terminal NudC family domain, followed by a coil-coiled domain and p23 domain connected by a flexible linker, and two α-helices at its C-terminus (Figure 4). Studies on NudC family of proteins focused on their cochaperone and intrinsic chaperone functions, as seen in NUDC and NUDCL2, which have been linked to act as co-chaperones in Hsp90-related refolding of client proteins such as the glucocorticoid receptor and cohesin, respectively (86, 87). NUDCD3 itself has been implicated in maintaining dynein intermediate chain stability and cellular viability in knockdown studies, while its overexpression has been associated with cytokinesis defects(88, 89). NUDCD3 was also reported by Taipale et al. among the RAG1 interactome in a LUMIER screen of potential client proteins shared with Hsp90 (90). The same screen highlighted a tendency for NUDCD3 to interact with proteins containing kelch-like domains, which RAG2 contains (90, 91).

**Figure 5:**
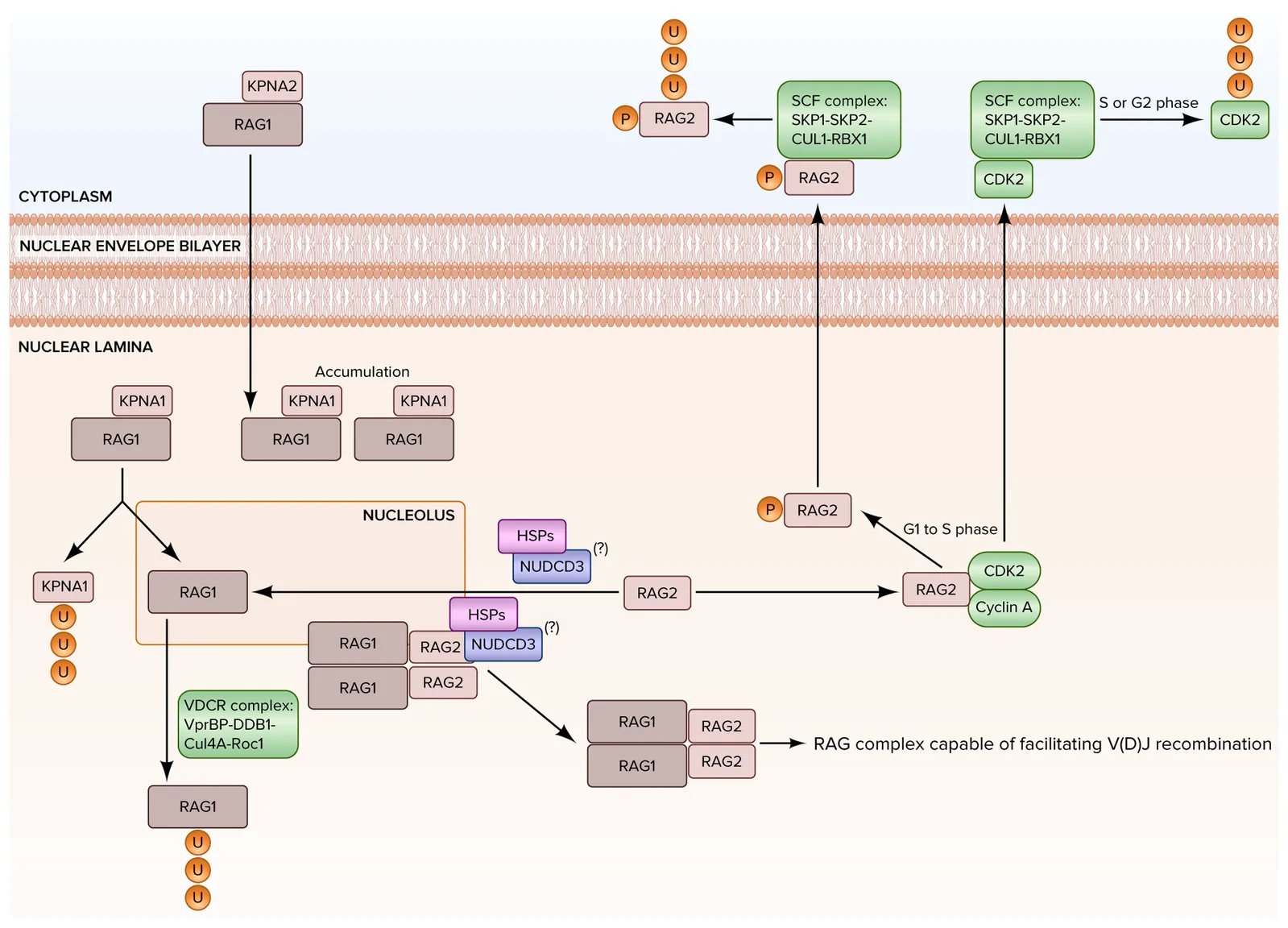
Cartoon representation of RAG trafficking and turnover in developing lymphocytes. Created with Biorender

In NUDCD3-deficient patient cells bearing the presumed founder mutation G52D, RAG1 becomes pathologically sequestered within nucleoli, preventing the RAG complex from initiating recombination and leading to a profound block in T and B cell development that phenotypically resembles RAG deficiency (84). Since our original report, two further immunodeficient patients with novel biallelic hypomorphic variants in NUDCD3 have recently been identified (Table S3). One missense variant (p.H313P) was identified in homozygosity in an infant with T-B- SCID while the other 2 variants (p.H19Q and Q302*) were found in compound heterozygosity in an older child with CID (Fabian Hauck, personal communication). While protein expression was profoundly reduced in patient cells, V(D)J recombination activity has yet to be formally tested. These findings suggest the testable hypothesis that NUDCD3 acts as a molecular co-chaperone facilitating the stabilization, trafficking, and/or subnuclear dynamics of the RAG recombinase (Figure 5). NUDCD3 deficiency represents a new molecular mechanism for recombination failure, highlighting novel aspects of RAG1 biology that are potentially targetable for therapeutic benefit (84).

Together, defects in RAG1, RAG2, and now NUDCD3 highlight the exquisite sensitivity of T cell development to perturbations in the cleavage stage of V(D)J recombination. These conditions span from absent T-cell development to severe immune dysregulation, underscoring how even subtle alterations in recombinase activity shape T and B cell repertoire and clinical phenotype.

#### Ligation

The non-homologous end joining (NHEJ) pathway is essential for repairing radiation-induced DNA double-strand breaks and for processing programmed lesions generated during V(D)J recombination. Germline defects in core NHEJ factors cause marked radiosensitivity and variably penetrant immune dysfunction, often accompanied by microcephaly, growth impairment, or developmental delay (92, 93). These disorders confer an increased risk of malignancy, both lymphoid and extrahematopoietic, as well as heightened sensitivity to toxicity associated with both irradiation and chemotherapy(92, 94).

Not all NHEJ components are clearly linked to human diseases, such as the auxiliary factor PAXX (68) and the Ku heterodimer XRCC5/XRCC6 (95), nor consistently to immunodeficiency, as seen in XRCC4 deficiency (68, 96, 97). By contrast, variants in *DCLRE1C, LIG4, NHEJ1* (XLF/Cernunnos) and *PRKDC* are associated with broader and more complex clinical phenotypes.

#### Ligation: End processing defects in TCR coding joint formation

**Figure 6:**
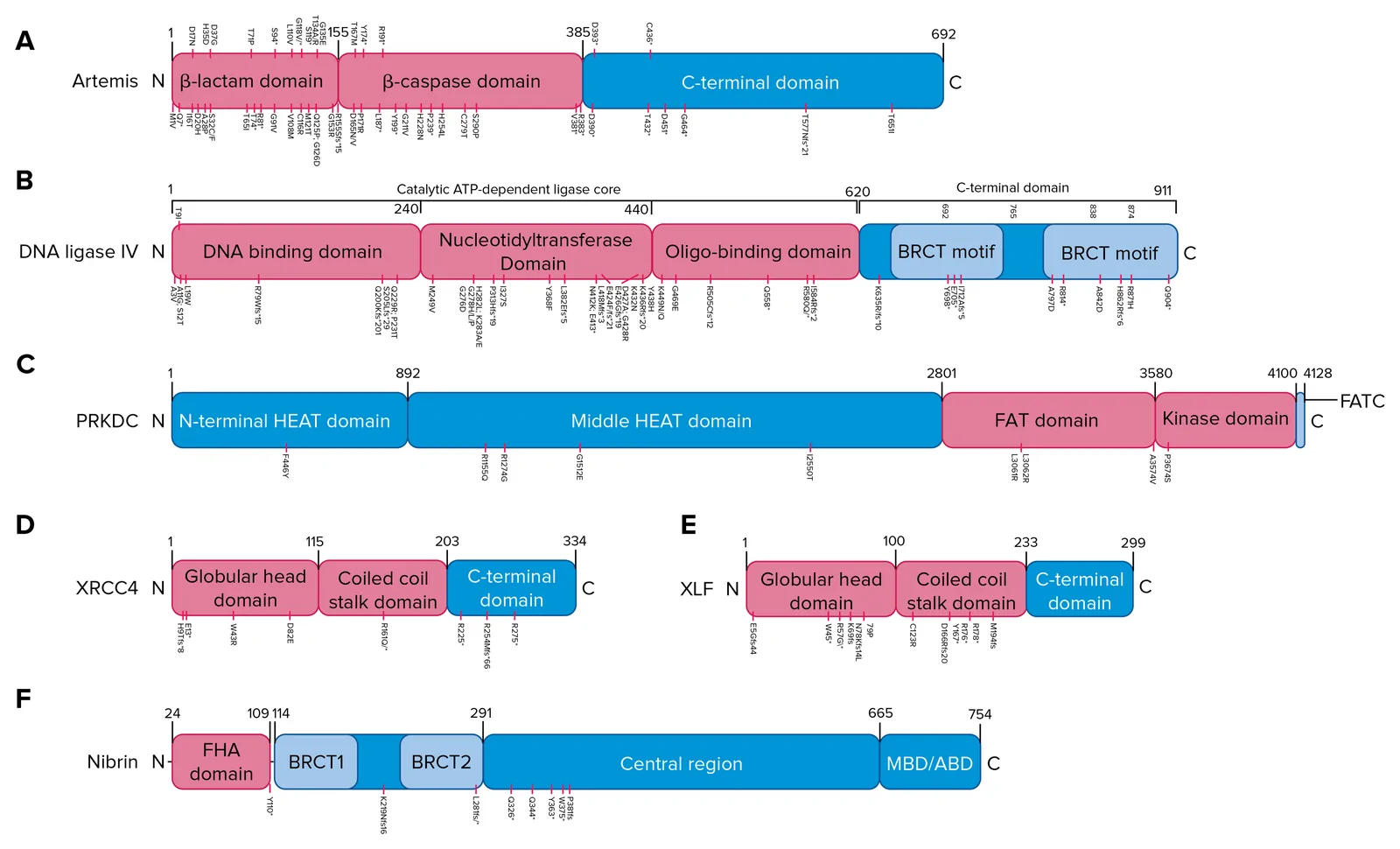
Domain structure and distribution of pathogenic variants in proteins involved in sensing and repair of DNA double strand breaks created during V(D)J recombination. BRCT, BRCA1 carboxyl terminus; HEAT, Huntingtin-Elongation Factor 3-PP2A-TOR1 repeat domain; FAT, FRAP–ATM–TRRAP domain. Based on (171–175). Created with Biorender

Artemis, a nuclease essential for opening DNA hairpins during V(D)J recombination and repairing DNA double-strand breaks, ensures proper T- and B-cell development. Biallelic pathogenic variants in *DCLRE1C*, encoding Artemis, give rise to diverse clinical manifestations (Figure 6; Table S4) (98–100). While null activity typically causes T–B–NK+ SCID, often accompanied by radiosensitivity (101, 102), hypomorphic alleles result in leaky SCID, sometimes complicated by EBV-driven lymphoma (103), granulomatous disease (104), inflammatory bowel disease (105), or hyper-IgM phenotypes (106).

#### Ligation: c-NHEJ pathway defects with systemic radiosensitivity

DNA-PKcs, encoded by the *PRKDC* gene, is the only active kinase within the NHEJ machinery (107). Recruited by Ku70/Ku80 to DNA breaks, it activates Artemis and it is involved in telomere maintenance, cell-cycle regulation and stress responses (107, 108). Reflecting its pleiotropic role, *PRKDC* variants cause a spectrum of conditions ranging from RS-SCID (109) to atypical SCID with prominent autoimmunity (110, 111) (Figure 6; Table S5). In addition, heterozygous *PRKDC* variants have been recently linked to IgG4-related disease, suggesting a potential genetic contribution to multifactorial pathogenesis of this condition (112). Unlike other NHEJ defects, only a few cases involve profound neurological manifestations (113) or congenital organ malformations (114).

The *LIG4* gene (Figure 6; Table S6) encodes for a core NHEJ enzyme, the DNA ligase IV, which with XRCC4 seals DNA double-strand breaks to restore genomic integrity (115, 116). Variants impairing its function have been reported across multiple series, illustrating an heterogeneous range of clinical manifestations, most notably, the “LIG4 syndrome”, characterized by skeletal anomalies, distinctive facial features, variable immunological impairment and increased susceptibility to lymphoma and bone marrow failure (117–121).

XRCC4 (Figure 6; Table S7) stabilizes DNA ligase IV during the final ligation steps of NHEJ. Biallelic pathogenic variants cause primordial dwarfism with severe microcephaly, short stature and neurodevelopmental delay, typically without immunodeficiency (97). Nevertheless, more recent functional studies have revealed a compromised TCR repertoire in lymphocytes of affected individuals (28, 122, 123).

XLF, encoded by *NHEJ1*, shows a central role in NHEJ, mainly relating to the LIG4/XRCC4 complex (124, 125). Despite its well-established task in NHEJ, the immunological effects of XLF deficiency are surprisingly variable, ranging from progressive T and predominantly B-cell lymphopenia to autoimmune manifestations (124, 126)(Figure 6; Table S8). As in LIG4 and NBN deficiencies, developmental defects and microcephaly have been reported (127).

Variants in core NHEJ genes, particularly *NHEJ1, LIG4*, and *XRCC4*, have been reported to skew the T cell receptor (TCR) repertoire. These alterations lead to recurrent infections, autoimmunity, immune dysregulation, and increased malignancy risk due to reduced receptor diversity, oligoclonality, and impaired lymphocyte function. Notably, disease manifestations and repertoire defects can occur even without profound lymphopenia, underscoring the role of TCR/BCR diversity, rather than lymphocyte count, in protective immunity (28;123;127–130). Beyond immunological dysfunction, NHEJ gene variants also affect organogenesis, growth, and neurodevelopment, highlighting the essential role of intact DNA repair machinery beyond V(D)J recombination (68).

#### Genome maintenance and checkpoint genes where thymocytes are uniquely vulnerable

Interestingly, disorders of DSB repair such as A-T often incur only mild immunodeficiency despite an apparently central role in V(D)J recombination (73). A-T is a rare autosomal recessive disorder leading to the development of telangiectasia, cerebellar ataxia and neurodegeneration as well as T and B cell immunodeficiency and predisposition to cancer, especially of lymphoid origin. While immunodeficiency may be detectable early in life including through TREC screening, the first clinical signs of the disease are usually cerebellar ataxia and neurodegeneration at the toddler stage (131). The apparent contradiction between ATM’s central role in orchestrating DSB repair and the mild and variable immunodeficiency in A-T is the subject of a recent review highlighting the redundancy between many of the mediators and processes carrying out NHEJ and DSB, specifically those involved in end tethering and coding hairpin resolution (73). Despite this, patients with A-T do show reduced TCR and antibody diversity, lymphopenia and fewer naïve cells, supporting a role for ATM in repairing RAG-induced DSB and V(D)J recombination (132). Interestingly, this picture includes a relatively increased proportion of γδ T lymphocytes (133, 134), one possible explanation being that ATM is only required once the γ and δ genes have partially rearranged at DN2 and before subsequent β rearrangement at DN3. However, the absolute number of γδ T cells in ATM deficient mice *is* reduced (133). Furthermore, thymic organoid culture experiments using murine *Atm*^-/-^ bone marrow progenitors show decreased (or delayed) appearance of TCRγδ and TCRβ expressing T cells (133). Similarly, malignancies (mostly T cell leukaemias and B cell lymphomas), common in A-T, show translocations in the TCR and immunoglobulin loci and these do not spare the *TRD* locus (135). Perhaps most interestingly, concurrent absence of RAG1 completely prevents the development of lymphoid tumours in ATM-deficient mice, suggesting that lymphoid oncogenesis in the absence of ATM is driven by V(D)J recombination (136). Consistent with an important role in CSR, patients typically have IgG and IgA deficiency and can occasionally present as hyper-IgM syndrome (131). Patients are typically not susceptible to the opportunistic infections seen in RAG deficiency but do develop sinopulmonary infections, with neurological complications such as dysphagia and impaired mobility increasing the chance of evolution to bronchiectasis and chronic lung disease (131).

Exactly how ATM deficiency leads to neurodegeneration is unclear although current understanding is that certain cerebellar neurons are particularly sensitive to the accumulation of DSBs (135). Supportive care is the mainstay of treatment although allogeneic HSCT using modified conditioning has been reported in a small cohort of A-T cases (associated with significant complications and no effect on neurological progression) (137).

Reflecting their function alongside ATM in DSB repair, the clinical phenotypes of MRE11 and NBN deficiency (Nijmegen Breakage syndrome, NBS; Figure 6, Table S9) overlap significantly with A-T, especially in the case of MRE11 (138). The phenotype of NBS is broader and includes craniofacial features such as a receding forehead and epicanthic folds as well as microcephaly, clinodactyly, syndactyly, anal atresia, and hydronephrosis; these may dominate the initial presentation (135). Absence of NBN leads to failure of both ATM *and* CtIP recruitment to DSBs resulting in impaired NHEJ *and* HR (135). Clinically this may manifest as immunodeficiency and/or immune dysregulation but a common presentation is with lymphoid malignancy in a child with a history of microcephaly and short stature. A few cases of an NBS-like disorder due to biallelic variants in *RAD50* have been reported with a cellular phenotype characterized by chromosomal instability, radiosensitivity, impaired signalling through ATM and impaired cell cycle checkpoint activation (139–142).

RNF168 deficiency leads to RIDDLE syndrome, a disorder characterized by radiosensitivity, dysmorphic features, learning difficulties and immunodeficiency, specifically impaired CSR (79, 143). Patients do not share the typical neurological features seen in patients with A-T and NBS. The ubiquitin ligase RNF168 is essential for the recruitment of 53BP1 to sites of DNA damage and interestingly, mice deficient in 53BP1 (144) share a specific defect of CSR with preserved V(D)J recombination and SHM. Finally, patients with CtIP deficiency suffer poor growth, developmental delay and skeletal abnormalities as well as being at risk of developing malignancies but are not reported to have any immunodeficiency perhaps reflecting the importance of NHEJ versus HR in repairing DSB induced during V(D)J recombination and CSR(145).

### Current therapeutic strategies and experimental therapies

#### Recognising T cell recombination defects; newborn screening and clinical presentation

The introduction of newborn screening for SCID in many countries enables the early identification of patients with T cell lymphopenia, pre-emptive institution of protective maneuvers and diagnostic work-up, and prompt referral for potentially curative therapy such as HSCT(146). The benefits of early HSCT in SCID are well-known but a recent longitudinal study in the USA demonstrated that affected infants identified by newborn screening had improved outcomes after HSCT, attributed to younger age and freedom from infection (3). Newborn screening uses the identification of stable non-replicating DNA episomes known as receptor excision circles generated during V(D)J recombination (TRECs in T cells and KRECs in B cells). Once identified as having low TRECs, patients have confirmatory lymphocyte subsets and further diagnostic work-up as appropriate (146).

#### Functional readouts: repertoire sequencing and *ex vivo* recombination assays

The diagnostic focus following confirmation of significant T cell lymphopenia or SCID is now on genetic screening, which in the UK is generally achieved by whole genome or whole exome sequencing. The results of adjunctive testing are also important, however, particularly in those infants who do not obtain a molecular diagnosis. Residual T cells should be assessed for materno-fetal engraftment and oligoclonality of the TCR repertoire, either consistent with SCID. Clinical TCR repertoire analysis is still typically done using flow cytometry of different V□ chains across CD4 and CD8 T cell subsets. The TCR repertoire, specifically the TCR V□ CDR3, can also be assessed using spectratyping or high-throughput sequencing, and these assays are part of the Primary Immune Deficiency Treatment Consortium (PIDTC) 2022 definition of SCID (147). CDR3 is the most variable part of the TCR and abnormalities of CDR3 length distribution tend to parallel the severity of associated immunodeficiency (148). The iterative nature of V(D)J recombination at the TRA locus also provides diagnostic clues, since its reduced efficiency prevents the incorporation of distal gene segments such as Vα7.2 – this is assessed as part of the PROMIDISα (PROximal-MIddle-DIStal T-cell receptor alpha) assay (28). The diagnostic role of T cell proliferation studies has been de-emphasised in SCID and T cell lymphopenia as the few T cells present can have normal proliferative responses to PHA (149).

Importantly, the differential diagnosis for isolated T cell lymphopenia includes congenital thymic defects which are not amenable to HSCT. In the absence of a molecular diagnosis for T-B+NK+ SCID, a role is thus increasingly recognized for *ex vivo* artificial thymic organoid assays to establish the T cell differentiation potential of HSC, albeit this remains a research test and results need to be interpreted with caution (150–152). In addition, assessment of fibroblast radiosensivity can aid the identification of patients with DNA repair disorders, who require reduced intensity conditioning to avoid toxicity (153). Some of these investigations take weeks to result and thus are best initiated in parallel with other molecular diagnostic studies to inform treatment decisions. Finally, research assays to characterise recombination activity *ex vivo* can be useful if novel variants in unknown genes are identified without other clear evidence of pathogenicity (83).

#### Curative therapy for T cell disorders

SCID was the first disorder to be treated successfully by allogeneic HSCT (154) and transplantation remains the cornerstone of curative treatment. Transplant outcomes in SCID are typically better than for non-SCID IEI and have improved dramatically over time as a result of earlier diagnosis, enabling prompt isolation and treatment before patients develop infections (3, 155). Nonetheless, disorders of V(D)J recombination show relatively poorer outcomes both in the short and long term and this is particularly marked in children with non-SCID presentations featuring chronic infection and autoimmunity (156).

Conventional myeloablative conditioning (MAC) in this cohort often results in catastrophic tissue toxicity, characterized by multi-organ failure, severe growth retardation and dental aplasia as well as increased rates of post-transplant autoimmunity (157–159). To mitigate these risks, clinical protocols have transitioned toward modified Fanconi-type conditioning regimens with Fludarabine and Cyclophosphamide and Reduced-Intensity Conditioning (RIC), utilizing pharmacokinetic-monitored low-dose busulfan or treosulfan to balance engraftment with long-term tolerability(160). Furthermore, the emergence of non-genotoxic conditioning via anti-CD117 monoclonal antibodies (e.g., briquilimab) represents a paradigm shift, potentially allowing for robust hematopoietic stem cell engraftment while completely bypassing the DNA-damaging effects inherent to traditional chemotherapy(161).

For some forms of SCID, gene therapy is available. Initially pioneered for ADA SCID and X-linked SCID, gene therapy requires less conditioning than HSCT and avoids graft-versus-host disease (162). Recent and ongoing studies using self-inactivating lentiviral vectors for gene addition show considerable promise in disorders of V(D)J recombination such as Artemis and RAG1 deficiency (163) (ClinicalTrials.gov identifier: NCT04797260). There have, however, been significant problems with the financial and regulatory models for delivery of gene therapy, resulting in disinvestment by pharmaceutical companies (164). Correction of the specific gene defect using CRISPR-based genome editing represents a promising alternative strategy (164). While loss of gene marking over time has complicated gene therapy for a number of IEI, this might be less problematic in the case of V(D)J recombination defects, in which correction is required very briefly during lymphocyte development. Indeed, the potential for genotoxicity as a result of developmentally inappropriate RAG expression might rather inspire additional caution regarding gene addition as opposed to gene correction, or even hypothetical drug therapies that might transiently rescue hypomorphic RAG activity.

### What remains unknown about V(D)J recombination: future directions, research priorities and concluding remarks

Many of the individual steps of V(D)J recombination, from DNA cleavage to ligation and repair, are well-characterised. IEI causing SCID and OS have demonstrated the essential roles of RAG, NUDCD3, Ligase 4 and Artemis for V(D)J recombination but a proportion of patients with similar presentations still lacks a molecular diagnosis. It is likely that at least some bear yet to be discovered pathogenic variants in genes or non-coding parts of the genome that are essential to V(D)J recombination. Recent research has focused on the importance of subcellular localization of and access to chromatin by RAG both of which appear to be mediated by non-core domains of the proteins. The exact molecular mechanisms that regulate the RAG2-dependent nucleolar egress of RAG1, however, are poorly understood and likely involve further chaperone proteins over and above NUDCD3 (84).

OS and CID can also result from variants in a number of genes involved in DNA synthesis, including polymerase delta 1 (*POLD1*), polymerase delta 3 (*POLD3*) and polymerase epsilon 2 (*POLE2)* while variants of the functionally related DNA primase small subunit (*PRIM1*) instead present with B cell lymphopenia and a/hypogammaglobulinemia (165). In fact, the T cell compartment in X-linked reticulate pigmentary disorder and van Esch-O’Driscoll syndrome due to variants in polymerase alpha 1 remains largely uncharacterized (166). Clearly, DNA synthesis is essential for successful NHEJ and V(D)J recombination but a deeper understanding of genotype-phenotype relationships in these known and novel disorders of the DNA replisome may indicate specific roles in V(D)J recombination.

Finally, the expanding phenotypic spectrum of affected individuals emphasises the importance of continuous evaluation and close collaboration in a small field looking after patients with ultrarare disorders. Identifying SCID-causative variants and understanding their pathomechanism remain both a clinical and scientific imperative, enabling us to initiate early, effective and targeted therapy as well as further our understanding of the molecular machinery essential for V(D)J recombination and thus for adaptive immunity.

## Additional information

Supplemental Tables S1-S9: https://doi.org/10.6084/m9.figshare.31843939
